# Calcium flux-independent NMDA receptor activity is required for A*β* oligomer-induced synaptic loss

**DOI:** 10.1038/cddis.2015.160

**Published:** 2015-06-18

**Authors:** J H Birnbaum, J Bali, L Rajendran, R M Nitsch, C Tackenberg

**Affiliations:** 1 Division of Psychiatry Research, University of Zurich, Schlieren, Switzerland; 2 Graduate Program of the Zurich Neuroscience Center, University of Zurich, Schlieren, Switzerland; 3 Systems and Cell Biology of Neurodegeneration, University of Zurich, Schlieren, Switzerland

## Abstract

Synaptic loss is one of the major features of Alzheimer's disease (AD) and correlates with the degree of dementia. *N*-methyl-d-aspartate receptors (NMDARs) have been shown to mediate downstream effects of the *β*-amyloid peptide (A*β*) in AD models. NMDARs can trigger intracellular cascades via Ca^2+^ entry, however, also Ca^2+^-independent (metabotropic) functions of NMDARs have been described. We aimed to determine whether ionotropic or metabotropic NMDAR signaling is required for the induction of synaptic loss by A*β*. We show that endogenous A*β* as well as exogenously added synthetic A*β* oligomers induced dendritic spine loss and reductions in pre- and postsynaptic protein levels in hippocampal slice cultures. Synaptic alterations were mitigated by blocking glutamate binding to NMDARs using NMDAR antagonist APV, but not by preventing ion flux with Ca^2+^ chelator BAPTA or open-channel blockers MK-801 or memantine. A*β* increased the activity of p38 MAPK, a kinase involved in long-term depression and inhibition of p38 MAPK abolished the loss of dendritic spines. A*β*-induced increase of p38 MAPK activity was prevented by APV but not by BAPTA, MK-801 or memantine treatment highlighting the role of glutamate binding to NMDARs but not Ca^2+^ flux for synaptic degeneration by A*β*. We further show that treatment with the G protein inhibitor pertussis toxin (PTX) did not prevent dendritic spine loss in the presence of A*β* oligomers. Our data suggest that A*β* induces the activation of p38 MAPK and subsequent synaptic loss through Ca^2+^ flux- and G protein-independent mechanisms.

Alzheimer's disease (AD) is clinically characterized by cognitive impairments caused by massive neuronal degeneration and synaptic loss. The reduction in synapse numbers is the best neuropathological correlate to the degree of dementia in AD.^1^ Besides synaptic alterations, the levels of soluble oligomeric forms of *β*-amyloid peptide (A*β*) but not plaques correlates best with memory loss in AD.^2^ Accumulating evidence indicates that transgenically produced A*β* or the treatment with A*β* oligomers decrease dendritic spine density,^3, 4, 5, 6^ impair long-term potentiation (LTP),^7^ facilitate long-term depression (LTD)^8^ and induce aberrant spine morphology.^5, 9^

Although the signaling cascades coupling A*β* with synaptic degeneration are incompletely understood, experimental evidence suggests an essential role for *N*-methyl-d-aspartate receptors (NMDARs). Oligomeric A*β* can bind to dendritic spines and treatment with NMDAR antibodies abolishes A*β* binding.^10^ Pharmacological inhibition of NMDAR activity also mitigates the pathological effect of A*β* on synapses.^4, 5, 6, 11^ NMDARs are ionotropic receptors permeable for cations and controlled by a voltage-dependent Mg^2+^ block that is removed after membrane depolarization by *α*-amino-3-hydroxy-5-methyl-4-isoxazolepropionic acid receptors (AMPARs). Upon glutamate binding to the NR2 subunit of NMDARs, cations including Ca^2+^ enter the cell. It has been thought for a long time that the levels of Ca^2+^ influx through NMDARs determine the induction of either LTP (high Ca^2+^ influx or LTD (mild Ca^2+^ influx).^12^ Nevertheless, a recent study showed that Ca^2+^ flux is not essential for the induction of NMDAR-LTD, whereas glutamate binding to the receptor is required.^13^ NMDAR signaling independently of ion flux has already been proposed to regulate NMDAR phosphorylation and endocytosis.^14, 15^ Further, the subunit switch between NR2B and NR2A NMDARs is driven by glutamate in the absence of NMDAR currents.^16^ However, the role of ion flux for synaptic loss in AD still remains to be elucidated.

We show that the A*β*-induced pre- and postsynaptic loss is mediated by glutamate binding to NMDARs, independent of ion influx.

## Results

**Figure 1:**
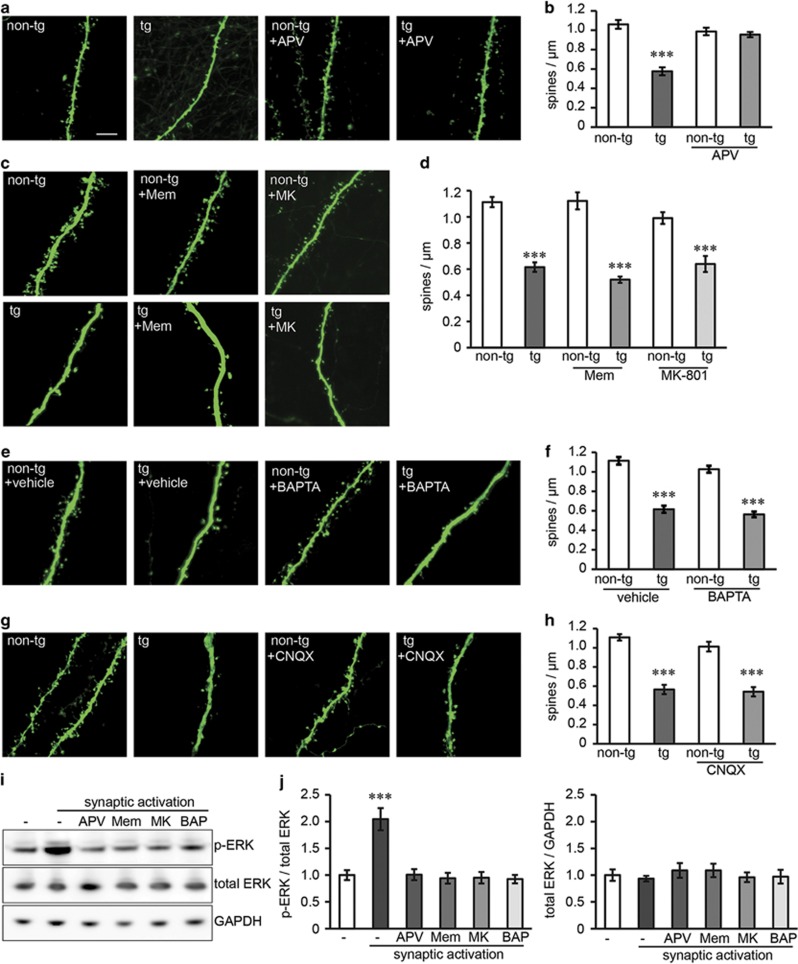
Blocking glutamate binding to NMDARs but not Ca^2+^ influx prevents dendritic spine loss in arcA*β*-transgenic slice cultures. (**a**) Confocal images of dendrites from CA1 neurons in the stratum radiatum of non-transgenic and arcA*β*-transgenic hippocampal slice cultures treated with NMDAR antagonist APV (100 *μ*M). Scale bar: 5 *μ*m. (**b**) APV treatment reverses the dendritic spine loss in arcA*β*-transgenic cultures. *n*=10–13. (**c**) Confocal images of non-transgenic and arcA*β*-transgenic cultures treated with NMDAR open-channel blocker memantine (1 *μ*M) or MK-801 (30 *μ*M). (**d**) Neither memantine nor MK-801 treatment reverses spine loss. *n*=11–13. (**e**) Confocal images of cultures treated with Ca^2+^ chelator BAPTA (2 mM) or vehicle (BAPTA solvent NaHCO_3_). (**f**) BAPTA treatment does not affect spine loss in transgenic cultures. (**g**) Confocal images of cultures treated with AMPAR antagonist CNQX (10 *μ*M). (**h**) CNQX treatment does not affect spine loss in transgenic cultures. *n*=11–15. (**i**) Western blot of lysate from non-transgenic cultures after synaptic activation—in the presence of the reagents used above—showing phosphorylated and total ERK levels. (**j**) APV (100 *μ*M), memantine (1 *μ*M), MK-801 (30 *μ*M) and BAPTA (2 mM) pre-treatment prevent ERK phosphorylation after synaptic activation. All values are shown as mean±S.E.M.; ***P<0.001; two-tailed unpaired Student's *t*-test; significances show difference to the respective non-transgenic control (**b**–**h**) or to non-activated cultures (**j**). non-tg, non-transgenic; tg, arcA*β* transgenic; Mem, memantine; BAP, BAPTA; MK, MK-801; p-ERK, phospho-ERK

To determine the mechanisms of synaptic loss by A*β*, we cultured hippocampal slices from arcA*β*-transgenic mice and infected them with neurotropic Sindbis virus expressing EGFP to visualize single neurons. Neurons in transgenic slices showed reduced dendritic spine densities. Treatment with the NMDAR antagonist D-APV, which blocks the glutamate-binding sites, completely abolished spine loss (Figures 1a and b). This is in agreement with previous findings that the glutamate-binding site antagonist CPP rescued spine loss in APP-transgenic cultures.^5^ As NMDAR signaling has been reported to be mediated through Ca^2+^ influx, we sought to determine the role of Ca^2+^ influx for A*β*-induced loss of dendritic spines. To this end, slices were treated with NMDAR open-channel blockers memantine or MK-801 (Figure 1c) or with Ca^2+^ chelator 1,2-bis(2-aminophenoxy)ethane-*N*,*N*,*N*',*N*'-tetraacetic acid (BAPTA; Figure 1e). Neither memantine nor MK-801 nor BAPTA treatment restored spine density in transgenic cultures (Figures 1d and f). Ca^2+^ flux through NMDARs requires the removal of the Mg^2+^ block within the receptor pore, which is achieved upon membrane depolarization by AMPARs. To analyze the involvement of AMPARs, we treated cultures with AMPAR antagonist CNQX, but did not observe any effect on spine numbers in transgenic cultures (Figures 1g and h). Concentrations of inhibitors were chosen according to previous reports demonstrating highest degree of specificity and/or therapeutic relevance (APV and MK-801;^17^ memantine^18, 19^).

To ensure that memantine, MK-801 and BAPTA preparations at the used concentrations are functional and can block NMDARs and the entry of Ca^2+^, despite having no protective effect, we performed synaptic activation experiments in the presence of these compounds. Synaptic activation induced phosphorylation of extracellular signal regulated kinases (phospho-ERK, p-ERK), which is in agreement with previous studies^6, 20^ (Figure 1i). The presence of APV, memantine, MK-801 or BAPTA fully prevented ERK activation, confirming the functionality of these compounds (Figure 1j). Of note, memantine blocked synaptic activation although it has been described to preferentially block extrasynaptic over synaptic activity at the used concentration.^21^

As shown previously, spine loss in arcA*β*-transgenic cultures can be prevented in the presence of anti-A*β* antibodies,^6^ confirming that A*β* but not APP or any other cleavage product is responsible for the observed effects on spines. Hence, our data indicate that glutamate binding to NMDARs rather than Ca^2+^ flux mediates A*β*-induced dendritic spine loss.

**Figure 2:**
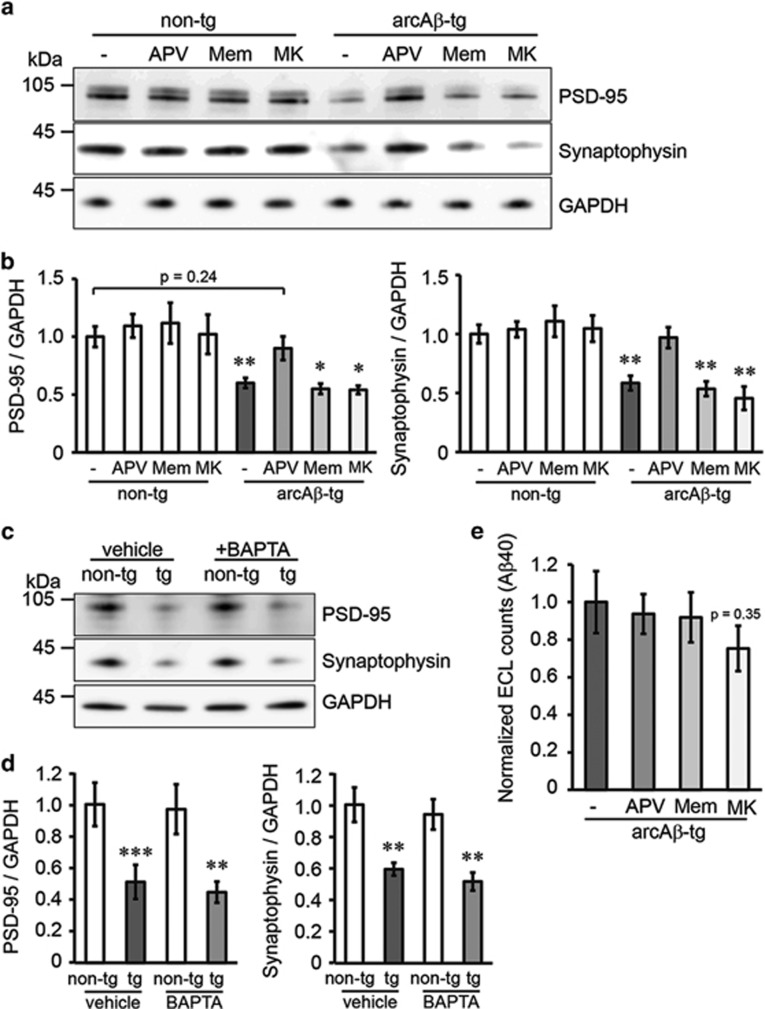
Blocking glutamate binding to NMDARs but not Ca^2+^ influx prevents the loss of pre- and postsynaptic markers in arcA*β*-transgenic cultures. (**a**) Representative western blot of cell lysates from non-transgenic or arcA*β*-transgenic cultures after treatment with NMDAR antagonists APV (100 *μ*M), memantine (1 *μ*M) or MK-801 (30 *μ*M). (**b**) Quantification of western blots. PSD-95 and synaptophysin levels are strongly reduced in arcA*β*-transgenic cultures. APV treatment restores PSD-95 and synaptophysin signals back to control levels, whereas memantine and MK-801 have no effect. *n*=6. (**c**) Representative western blot of cell lysates from non-transgenic or arcA*β*-transgenic slices after treatment with Ca^2+^ chelator BAPTA (2 mM) or vehicle (BAPTA solvent NaHCO3). (**d**) BAPTA treatment does not affect loss of synaptic proteins in transgenic cultures. *n*=6. (**e**) A*β*40 levels in the supernatant of arcA*β*-transgenic cultures after treatment with NMDAR antagonists measured by MSD. A*β*40 production is not influenced by any NMDAR antagonist. A*β* levels were corrected by protein levels from lysates. *n*=3. All values are shown as mean±S.E.M. (*P<0.05, **P<0.01, ***P<0.001; two-tailed unpaired Student's *t*-test; significances indicate differences to the respective non-transgenic control). non-tg, non-transgenic; tg, arcA*β*-transgenic; Mem, memantine; MK, MK-801

To confirm the morphological spine data, we determined whether A*β* also affects the levels of pre- and postsynaptic proteins and analyzed PSD-95 (postsynaptic) and synaptophysin (presynaptic) levels in lysates of non-transgenic and arcA*β*-transgenic cultures (Figure 2a). Compared with controls, the levels of both proteins were strongly reduced in transgenic cultures. Treatment with APV but not with memantine or MK-801 rescued the reduction of protein levels (Figure 2b). Likewise, treatment with BAPTA did not affect loss of synaptic proteins in transgenic cultures (Figures 2c and d).

Synaptic activity has been shown to increase the production of A*β* and, inversely, preventing synaptic activity can reduce A*β* production.^22^ To exclude that the protective effect of APV is simply based on A*β* reduction, we quantitatively measured the levels of A*β*40 in the supernatant of arcA*β*-transgenic cultures treated with the respective NMDAR antagonists (Figure 2e) under identical conditions as in the previous experiments. NMDAR antagonists did not significantly alter A*β* levels in the medium of transgenic cultures.

This indicates that, in addition to dendritic spine loss, reductions in pre- and postsynaptic protein levels are caused by NMDAR functions, independent of Ca^2+^ flux.

**Figure 3:**
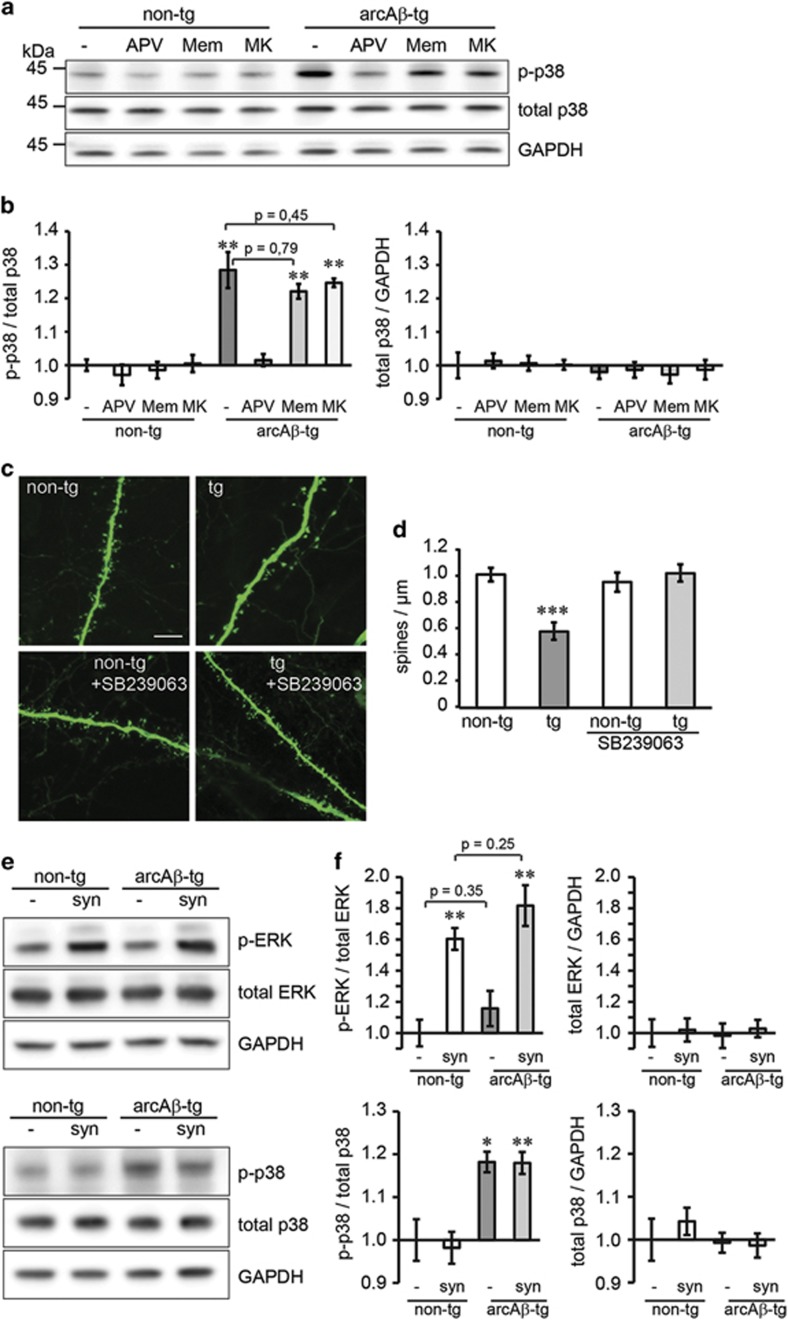
p38 MAPK is activated in arcA*β*-transgenic cultures and mediates spine loss. (**a**) Representative western blot showing phosphorylated (active) and total p38 MAPK in lysates non-transgenic or arcA*β*-transgenic slices after treatment with different NMDAR antagonists. (**b**) Quantification shows increased levels of phosphorylated p38 in arcA*β*-transgenic cultures. The increased amounts of phospho-p38 were reduced to control levels by APV (100 *μ*M) but not by memantine (1 *μ*M) or MK-801 (30 *μ*M) treatment. The non-transgenic untreated control was set to 1. *n*=6. (**c**) Confocal images of dendrites from CA1 neurons in the stratum radiatum of non-transgenic and arcA*β*-transgenic hippocampal slice cultures treated with p38 MAP kinase inhibitor SB239063 (20 *μ*M). Scale bar: 5 *μ*M. (**d**) SB239063 treatment reverses the dendritic spine loss in arcA*β*-transgenic cultures. *n*=14-16. (**e**) Representative western blot showing activated ERK (p-ERK) and activated p38 (p-p38) in non-transgenic or arcA*β*-transgenic slices after synaptic activation with bicuculline and 4-aminopyridine. (**f**) Quantification shows increased p-ERK levels after synaptic activation, independent of transgenic background. Synaptic activation does not affect p-p38 levels. *n*=5. All values are shown as mean±S.E.M. (*P<0.05, **P<0.01, ***P<0.001; two-tailed unpaired Student's *t*-test; significances indicate differences to the respective non-tg control; for p-ERK/total ERK significances indicate differences to the respective non-activated culture). non-tg, non-transgenic; tg, arcA*β* transgenic; Mem, memantine; MK, MK-801; p-ERK, phospho-ERK; p-p38, phospho-p38 MAPK; syn, synaptic activation

A recent study showed that activation of p38 MAPK is essential for Ca^2+^-independent metabotropic function of NMDARs.^13^ We analyzed whether p38 MAPK is also involved in the A*β* effects on synapses and examined the activity of p38 MAPK in lysates from non-transgenic and arcA*β*-transgenic cultures (Figure 3a). Increased levels of phosphorylated (active) p38 MAPK (p-p38) were observed in transgenic cultures. After treatment with APV, the levels of p-p38 were reduced to control levels, whereas memantine or MK-801 treatment had no effect (Figure 3b). To ascertain that active p38 MAPK mediates synaptic deficits caused by A*β*, we treated cultures with the p38 MAPK inhibitor SB239063. Treatment abolished spine loss in arcA*β*-transgenic cultures (Figures 3c and d). To examine whether a general increase in synaptic activity in transgenic cultures contributes to increased p-p38 MAPK levels, we treated non-transgenic and arcA*β*-transgenic cultures with bicuculline and 4-aminopyridine (Figures 3e and f). Synaptic activation increased levels of p-ERK. No difference in p-ERK levels were observed between transgenic and non-transgenic cultures, indicating that arcA*β-*transgenic slices display no general increase in synaptic activity. Further, synaptic activation does not affect the activity of p38 MAPK.

This data suggests that A*β* induces the activity of p38 MAPK, which mediates the loss of dendritic spines. This effect does not depend on Ca^2+^ influx or general synaptic activation.

**Figure 4:**
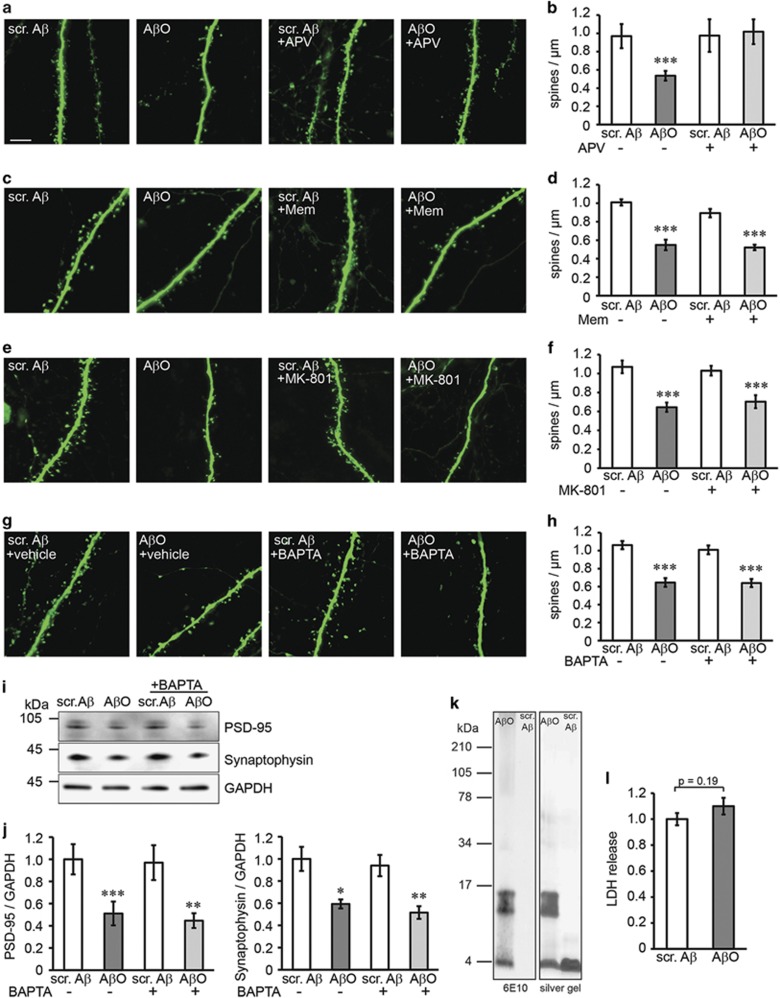
Oligomeric A*β*-induced synaptic loss is prevented by APV but not by memantine, MK-801 or BAPTA treatment. (**a**) Confocal images of dendrites from CA1 neurons in the stratum radiatum of non-transgenic slice cultures treated with oligomeric A*β* (500 nM) or scrambled A*β* (500 nM) and NMDAR antagonist APV (100 *μ*M). Scale bar: 5 *μ*M. (**b**) APV treatment prevents A*β* oligomer-induced dendritic spine loss. *n*=13–17. (**c**) Confocal images of non-transgenic cultures treated with oligomeric A*β* (500 nM) and NMDAR antagonist memantine (1 *μ*M). (**d**) Memantine treatment does not prevent spine loss. *n*=12–17. (**e**) Confocal images of cultures treated with oligomeric A*β* (500 nM) and NMDAR antagonist MK-801 (30 *μ*M). (**f**) MK-801 treatment does not prevent spine loss. *n*=13–15. (**g**) Non-transgenic cultures treated with A*β* oligomers (500 nM) and Ca^2+^ chelator BAPTA (2 mM) or vehicle (BAPTA solvent NaHCO_3_). (**h**) BAPTA does not prevent spine loss caused by oligomeric A*β*. *n*=11–15. (**i**) Representative western blot of cell lysates from slices after treatment with A*β* oligomers (500 nM) and Ca^2+^ chelator BAPTA (2 mM). (**j**) BAPTA does not prevent reduction in PSD-95 or synaptophysin levels after A*β* oligomer treatment. *n*=6. (**k**) SDS gel showing oligomeric A*β* preparations and scrambled A*β* after silver staining (right panel) and western blot stained with 6E10 antibody (left panel). Monomers, tri- and tetramers are observed in the oligomeric preparation, whereas scrambled A*β* only shows monomers. (**l**) LDH assay showing no toxicity of A*β* oligomer treatment (500 nM) compared with scrambled A*β*. *n*=6. Values are shown as mean±S.E.M. (*P<0.05, **P<0.01, ***P<0.001; two-tailed unpaired Student's *t*-test). Scr. A*β*, scrambled A*β*; A*β*O, oligomeric A*β*; Mem, memantine

Oligomeric A*β* is considered to be one of the main toxic A*β* species in the AD brain. So far, we used cultures from arcA*β*-transgenic mice to determine the effects of A*β* on synapses in the presence of other APP processing products (Figures 1–3). Although arcA*β* mice show early formation oligomeric A*β in vivo*,^23^ the role of oligomeric A*β* for our findings requires further investigations. To conclusively validate the role of A*β* oligomers, we treated non-transgenic cultures with defined preparation of A*β*42 oligomers at sublethal concentrations (Figure 4). Oligomer preparations contained mostly mono-, tri- and tetramers as determined by silver stained SDS gel and western blot (Figure 4k), which is in agreement with previous studies.^24, 25^ Scrambled A*β*, subjected to the same oligomerization protocol as A*β*42, did not aggregate, as expected. Treatment with A*β* oligomers but not scrambled A*β* reduced dendritic spine density to a similar extent as observed in transgenic cultures (compare Figure 4 and Figure 1). Confirming the transgenic data, only APV treatment (Figures 4a and b) but not memantine (Figures 4c and d), MK-801 (Figures 4e and f) or BAPTA (Figures 4g and h) prevented oligomer-induced spine loss. Oligomeric A*β* further reduced PSD-95 and synaptophysin levels, which could not be rescued by BAPTA treatment (Figures 4i and j). A*β* did not cause cell death at the used concentration (Figure 4l).

This indicates that oligomeric A*β*, similar to transgenically produced A*β*, exerts its toxic properties on synapses via NMDAR signaling, independent of Ca^2+^ influx.

**Figure 5:**
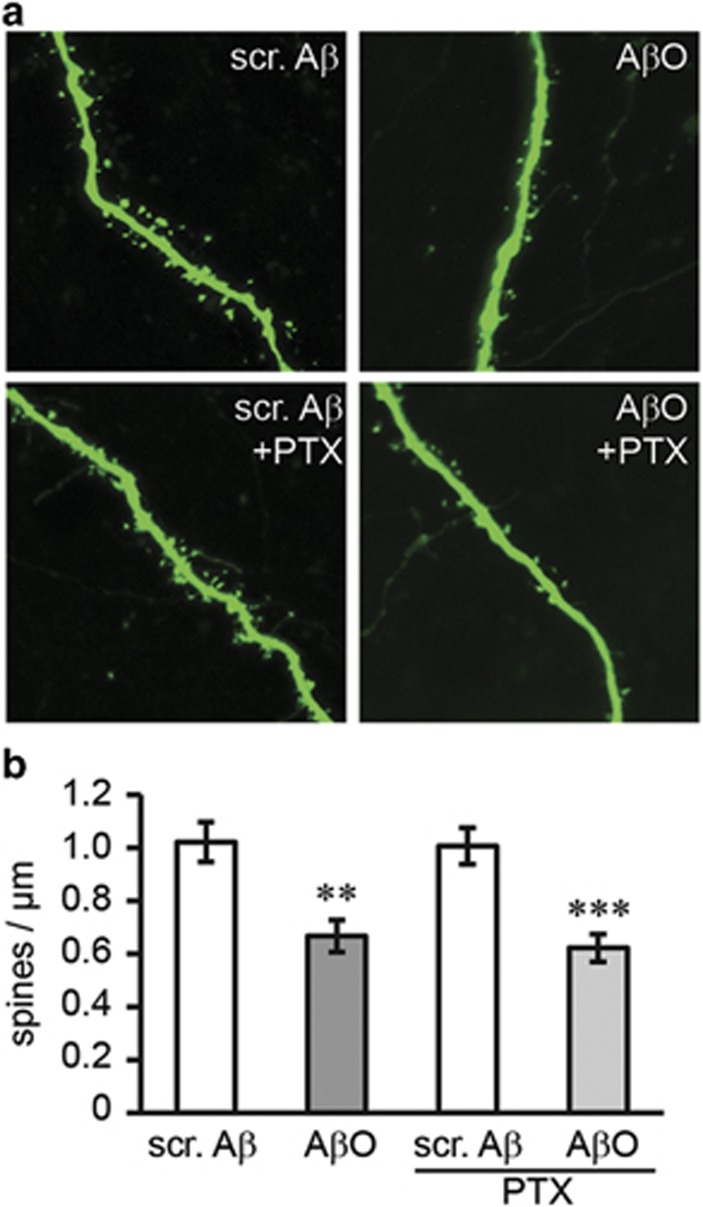
Oligomeric A*β*-induced synaptic loss is not prevented by treatment with PTX. (**a**) Confocal images of dendrites from CA1 neurons in the stratum radiatum of non-transgenic slice cultures treated with oligomeric A*β* (500 nM) or scrambled A*β* (500 nM) and G protein inhibitor PTX (500 ng/ml). (**b**) PTX treatment does not prevent A*β* oligomer-induced dendritic spine loss. *n*=12. Values are shown as mean±S.E.M. (**P<0.01, ***P<0.001; two-tailed unpaired Student's *t*-test). Scr. A*β,* scrambled A*β*; A*β*O, oligomeric A*β*; PTX, pertussis toxin

To determine whether Ca^2+^ flux-independent synaptic loss depends on G protein signaling, we treated slices with oligomeric A*β* and pertussis toxin (PTX), an inhibitor of the heterotrimeric G_i/o_ protein family, at concentrations described before in slice cultures.^26^ PTX administration did not prevent spine loss caused by A*β* (Figures 5a and b) suggesting that A*β*-induced synaptic loss does not require a PTX-sensitive subgroup of G proteins.

## Discussion

In this study, we have examined the role of Ca^2+^ flux for A*β*-induced loss of dendritic spines and pre- and postsynaptic proteins. Our data show that NMDAR-dependent ion flux is not required for synaptic loss, whereas binding of glutamate to the NMDAR is essential for coupling A*β* with synaptic degeneration.

NMDARs have been thought to signal exclusively ionotropic, regulating intracellular signaling via Ca^2+^ transmission. However, recent evidence indicates that NMDARs can signal metabotropically, that is, independent of ion flux. The group of Roberto Malinow showed that induction of NMDAR-LTD via activation of p38 MAPK is based on metabotropic signaling.^13^ Further, the induction of LTD by A*β* can occur in the absence of Ca^2+^ transmission.^17, 27^ Together, these data suggest that glutamate binding to NMDARs may induce a conformational change that subsequently activates intracellular signaling cascades even in the absence of Ca^2+^ flux. This possibly does not exclude an additional role of intracellular Ca^2+^, because the injection of Ca^2+^ chelators into neurons prevents LTD induction.^13, 28^ In agreement, oligomeric A*β* has been shown to increase intracellular Ca^2+^ levels by mobilizing Ca^2+^ from the ER rather than promoting influx of Ca^2+^ from the extracellular space.^29^

Our data show that the induction of specific metabotropic-like NMDAR signaling pathway by A*β*, which is not induced by general synaptic activation, causes downstream phosphorylation of p38 MAPK. Active p38 MAPK is key player in NMDAR- and mGluR-dependent LTD^30, 31^ and mediates AMPAR endocytosis.^32^ A study by Yang *et al.*^33^ described an intracellular pathway based on the co-activation of mGluR5 and NMDARs, also independent of Ca^2+^ flux. Further, mGLuR5 has been implicated in mediating toxic effects of A*β* at synapses.^34, 35^ Thus, a co-activation of mGluR5 and NMDARs may cause downstream activation of p38 MAPK followed by synaptic loss. However, treatment with the G protein inhibitor PTX did not prevent spine loss in our model, which renders the involvement of mGluRs unlikely.

In previous studies, we showed that caspase-3 and calcineurin are essential for the loss of spines by A*β*.^5, 6^ Caspase-3 can be activated by p38 MAPK.^36, 37^ Further, D'Amelio and colleagues reported caspase-3- and calcineurin-mediated synaptic dysfunction in APP-transgenic mice. Importantly, they observed that caspase-3-activated calcineurin by proteolytic cleavage in a Ca^2+^-independent manner, supporting our finding that A*β*-induced synaptic dysfunction can occur in the absence of Ca^2+^ flux.

An important finding in our study is the lack of synaptic protection by memantine as memantine is the only clinical approved NMDAR antagonist for treatment of AD patients. Memantine, at clinically relevant low *μ*M concentrations, is a low-affinity, uncompetitive open-channel blocker with a relatively high off-rate.^38^ High-affinity NMDAR antagonists may be toxic after long exposure due to block of synaptic transmission. However, memantine has been suggested to be more tolerable because of blocking mainly over-excitation of the receptor rather than its physiological activity. As the high off-rate of memantine could still allow Ca^2+^ influx into the cell, we confirmed the data using a second open-channel blocker MK-801 and the Ca^2+^ chelator BAPTA. Further, all used compounds could fully block ERK phosphorylation after synaptic activation. Interestingly, treatment of slices with even the high-affinity inhibitors D-APV or MK-801 or with BAPTA did not show any side effects on spines.

Despite having no protective effect for A*β*-induced synaptic loss in our study, memantine may be more beneficial with respect to other A*β* effects. A recent study showed that injection of low-molecular-weight (LMW) oligomers into mice caused persistent memory impairment and synaptic loss, whereas injection of high-molecular-weight (HMW) oligomers resulted in neuronal oxidative stress and reversible cognitive deficits but no synaptic loss. Memantine treatment could rescue only the effects of HMW but not LMW A*β* oligomers,^39^ further indicating that memantine may not be beneficial with respect to A*β*-induced synaptic alterations. However, memantine protected against the induction of oxidative stress by oligomeric A*β*^10^ and studies from our lab showed that memantine, at the concentration used in this study, prevented the increase in tau phosphorylation by A*β* (unpublished observations).

Because synaptic loss occurs early in the disease process,^40^ our data my contribute to explain why memantine is ineffective in treating early-staged mild AD patients.^41^ Together, our data establish a Ca^2+^ flux- and G protein-independent NMDAR signaling pathway coupling A*β* toxicity with p38 MAPK activation and synapse loss, suggesting pharmacological inhibition of this pathway as a potent mechanism to prevent A*β*-mediated early synaptic loss.

## Materials and Methods

### Chemicals/reagents

Cell culture reagents were purchased from Sigma (Schnelldorf, Germany) and Invitrogen (Basel, Switzerland). NMDA receptor antagonists D-APV (also called D-AP5, d-2-amino-5-phosphonovalerate; Batch No.:71), MK-801 ((5 S,10 R)-(+)-5-methyl-10,11-dihydro-5*H*-dibenzo[a,d]cyclohepten-5,10-imine maleate; Batch No.:8), memantine (3,5-dimethyl-tricyclo[3.3.1.13,7]decan-1-amine hydrochloride; Batch No.:9), Ca^2+^ chelator BAPTA (Batch No.:4), AMPA receptor antagonist CNQX (6-cyano-7-nitroquinoxaline-2,3-dione; Batch No.:33) were purchased from Tocris (Bristol, UK). PTX was purchased from List Biological Laboratories (Campbell, CA, USA).

### Hippocampal slice cultures

ArcA*β*-transgenic mice were obtained as described.^23^ All animal experiments were performed in accordance with the guidelines of the Swiss veterinary cantonal office. Hippocampal slice cultures were prepared and cultured as described.^42^ In short, 6–7-day-old transgenic and non-transgenic C57BL/6 mice were decapitated, brains were removed, hippocampi were isolated and cut into 400-*μ*m thick slices. Slices were cultured in culture medium (minimum essential medium Eagle with HEPES modification, 25% basal medium with Earle's modification, 25% heat-inactivated horse serum, 2 mM glutamine, 50 units per ml penicillin, 50 *μ*g/ml streptomycin, 0.6% glucose, pH 7.2). Culture medium was exchanged every second or third day. On DIV 11, culture medium was replaced by low-serum Nb-N2 medium (Neurobasal medium, 0.5% heat-inactivated horse serum, 2 mM glutamine, 50 units per ml penicillin, 50 *μ*g/ml streptomycin, 0.6% glucose, 1 × N2 supplement, pH 7.2) to ensure more defined condition during analysis. For spine analysis, slice cultures were infected with Sindbis virus expressing EGFP on DIV 12 in culture and fixed on DIV 15 with 4% paraformaldehyde/sucrose. For protein analysis, uninfected slices were lysed on DIV 15 in culture.

### Treatments

To determine inhibitor effects in transgenic cultures, slices were treated with respective inhibitors from DIV 11–15. To analyze effects of oligomeric A*β*, slices were treated with A*β* oligomers or scrambled A*β* from DIV 11–15. To assess the effects of inhibitors on cultures exposed to oligomeric A*β*, slices were treated with A*β* oligomers and the respective inhibitor in parallel from DIV 11–15. For treatment with PTX, slices were exposed to PTX from DIV 13–15.

### Dendritic spine analysis

To determine dendritic spine density, virus solution was diluted to achieve 1–10 infected neurons per slice to allow imaging of single dendritic fragments. Analysis of dendritic spine density was performed using Leica SP2 CLSM equipped with 63 × objective (NA: 1.2) and 488-nm Argon laser. Apical dendritic segments in CA1 stratum radiatum were imaged with size of 30 × 30 *μ*m (512 × 512 pixel, voxel size: 0.05813 × 0.05813 × 0.25 *μ*m). Image stacks were processed to maximum projections, and dendritic spine density was determined using ImageJ.

Spine imaging and counting were performed blinded (without the researcher knowing the mouse genotype or culture treatment).

### Synaptic activation protocol

Stimulation of synaptic activity was adapted from Tackenberg *et al.*^6^ Cultures were pretreated with APV, memantine, MK-801 or BAPTA for 12 h before activation. Then, cultures were exposed to neurobasal medium containing 1 mM 4-AP, 25 mM bicuculline and the respective inhibitor for 20 min. Control cultures were treated with neurobasal medium containing identical DMSO concentrations as above but devoid of 4-AP, bicuculline and inhibitors.

### Western blot

Cultured slices were harvested on DIV 15, sonicated in RIPA buffer (50 mM Tris-HCl, 150 mM NaCl, 2 mM EDTA, 1% NP-40, 0.5% deoxycholate and 0.1% SDS, pH 8.0) containing phosphatase inhibitor cocktails 1 and 2 (Sigma) and protease inhibitor cocktail (Roche, Basel, Switzerland) and centrifuged at 5000 g for 10 min at 4 °C. The supernatant was stored at −80 °C. Lysates were subjected to SDS-PAGE followed by immunoblotting using primary antibodies against PSD-95 and Synaptophysin (both Millipore, Billerica, MA, USA, 1 : 1000), phospho-p38 MAPK and p38 MAPK (both Cell Signaling, Danvers, MA, USA, 1 : 1000), 6E10 (Signet, Dedham, MA, USA, 1 : 500) and GAPDH (Biodesign, Saco, ME, USA, 1 : 5000). Band intensities were quantified with ImageJ.

### Silver staining

Oligomeric A*β*42 preparations were analyzed by SDS-PAGE. The gel was left overnight in fixing solution (40% EtOH, 10% acetic acid), sensitized in 0.017% sodium thiosulfate for 2 min, impregnated in 0.27% silver nitrate solution (including 0.37% formaldehyde) for 30 min and developed in 0.03 M sodium carbonate (supplemented with 0.15% formaldehyde and 0.02% sodium thiosulfate). The reaction was stopped in 3% glacial acid.

### A*β* oligomer preparations

Synthetic A*β*42 peptide was obtained from American peptide. Preparation of A*β*42 oligomers (Abeta-derived diffusible ligands) was carried out as previously described.^43^ Cold 1,1,1,3,3,3-hexafluro-2-propanol (HFIP) was added to A*β*42 peptide to a final concentration of 1 mM. HFIP was evaporated overnight, peptides dried for 10 min in a speedvac and stored at −80 °C. Peptides were resuspended in DMSO at 5 mM concentrations. Neurobasal medium without phenol red was added to achieve a peptide concentration of 100 *μ*M and incubated for 24 h at 4 °C. Higher aggregates, for example, fibrils were removed by centrifugation at 14 000 g for 10 minutes at 4 °C and the supernatant was used for experimental procedures. A*β*42 oligomer preparations were analyzed by silver staining and western blot for each experiment.

## Abbreviations

AD: Alzheimer's disease
A*β*: *β*-amyloid peptide
NMDAR: N-methyl-d-aspartate receptor
LTD: long-term-depression
PTX: Pertussis toxin
p-ERK: phospho-ERK

## References

[bib1] Terry RD, Masliah E, Salmon DP, Butters N, DeTeresa R, Hill R et al. Physical basis of cognitive alterations in Alzheimer's disease: synapse loss is the major correlate of cognitive impairment. Ann Neurol 1991; 30: 572–580. 1789684 10.1002/ana.410300410

[bib2] McLean CA, Cherny RA, Fraser FW, Fuller SJ, Smith MJ, Beyreuther K et al. Soluble pool of Abeta amyloid as a determinant of severity of neurodegeneration in Alzheimer's disease. Ann Neurol 1999; 46: 860–866. 10589538 10.1002/1531-8249(199912)46:6<860::aid-ana8>3.0.co;2-m

[bib3] Shrestha BR, Vitolo OV, Joshi P, Lordkipanidze T, Shelanski M, Dunaevsky A et al. Amyloid beta peptide adversely affects spine number and motility in hippocampal neurons. Mol Cell Neurosci 2006; 33: 274–282. 16962789 10.1016/j.mcn.2006.07.011

[bib4] Shankar GM, Bloodgood BL, Townsend M, Walsh DM, Selkoe DJ, Sabatini BL et al. Natural oligomers of the Alzheimer amyloid-beta protein induce reversible synapse loss by modulating an NMDA-type glutamate receptor-dependent signaling pathway. J Neurosci 2007; 27: 2866–2875. 17360908 10.1523/JNEUROSCI.4970-06.2007 PMC6672572

[bib5] Tackenberg C, Brandt R. Divergent pathways mediate spine alterations and cell death induced by amyloid-beta, wild-type tau, and R406W tau. J Neurosci 2009; 29: 14439–14450. 19923278 10.1523/JNEUROSCI.3590-09.2009 PMC6665808

[bib6] Tackenberg C, Grinschgl S, Trutzel A, Santuccione AC, Frey MC, Konietzko U et al. NMDA receptor subunit composition determines beta-amyloid-induced neurodegeneration and synaptic loss. Cell Death Dis 2013; 4: e608. 23618906 10.1038/cddis.2013.129 PMC3641351

[bib7] Shankar GM, Li S, Mehta TH, Garcia-Munoz A, Shepardson NE, Smith I et al. Amyloid-beta protein dimers isolated directly from Alzheimer's brains impair synaptic plasticity and memory. Nat Med 2008; 14: 837–842. 18568035 10.1038/nm1782 PMC2772133

[bib8] Li S, Hong S, Shepardson NE, Walsh DM, Shankar GM, Selkoe D et al. Soluble oligomers of amyloid Beta protein facilitate hippocampal long-term depression by disrupting neuronal glutamate uptake. Neuron 2009; 62: 788–801. 19555648 10.1016/j.neuron.2009.05.012 PMC2702854

[bib9] Lacor PN, Buniel MC, Furlow PW, Clemente AS, Velasco PT, Wood M et al. Abeta oligomer-induced aberrations in synapse composition, shape, and density provide a molecular basis for loss of connectivity in Alzheimer's disease. J Neurosci 2007; 27: 796–807. 17251419 10.1523/JNEUROSCI.3501-06.2007 PMC6672917

[bib10] De Felice FG, Velasco PT, Lambert MP, Viola K, Fernandez SJ, Ferreira ST et al. Abeta oligomers induce neuronal oxidative stress through an N-methyl-D-aspartate receptor-dependent mechanism that is blocked by the Alzheimer drug memantine. J Biol Chem 2007; 282: 11590–11601. 17308309 10.1074/jbc.M607483200

[bib11] Li S, Jin M, Koeglsperger T, Shepardson NE, Shankar GM, Selkoe DJ et al. Soluble Aß oligomers inhibit long-term potentiation through a mechanism involving excessive activation of extrasynaptic NR2B-containing NMDA receptors. J Neurosci 2011; 31: 6627–6638. 21543591 10.1523/JNEUROSCI.0203-11.2011 PMC3100898

[bib12] Chung C. NMDA receptor as a newly identified member of the metabotropic glutamate receptor family: clinical implications for neurodegenerative diseases. Mol Cells 2013; 36: 99–104. 23740429 10.1007/s10059-013-0113-y PMC3887951

[bib13] Nabavi S, Kessels HW, Alfonso S, Aow J, Fox R, Malinow R et al. Metabotropic NMDA receptor function is required for NMDA receptor-dependent long-term depression. Proc Natl Acad Sci USA 2013; 110: 4027–4032. 23431133 10.1073/pnas.1219454110 PMC3593861

[bib14] Vissel B, Krupp JJ, Heinemann S F, Westbrook GL. A use-dependent tyrosine dephosphorylation of NMDA receptors is independent of ion flux. Nat Neurosci 2001; 4: 587–596. 11369939 10.1038/88404

[bib15] Nong Y, Huang YQ, Ju W, Kalia LV, Ahmadian G, Wang YT et al. Glycine binding primes NMDA receptor internalization. Nature 2003; 422: 302–307. 12646920 10.1038/nature01497

[bib16] Barria A, Malinow R. Subunit-specific NMDA receptor trafficking to synapses. Neuron 2002; 35: 345–353. 12160751 10.1016/s0896-6273(02)00776-6

[bib17] Kessels HW, Nabavi S, Malinow R. Metabotropic NMDA receptor function is required for *β* -amyloid – induced synaptic depression. Proc Natl Acad Sci USA 2013; 110: 4033–4038. 23431156 10.1073/pnas.1219605110 PMC3593880

[bib18] Wesemann W, Sturm G, Fünfgeld EW. Distribution of metabolism of the potential anti-parkinson drug memantine in the human. J Neural Transm Suppl 1980; 16: 143–148. 6933219 10.1007/978-3-7091-8582-7_15

[bib19] Periclou A, Ventura D, Rao N, Abramowitz W. Pharmacokinetic study of memantine in healthy and renally impaired subjects. Clin Pharmacol Ther 2006; 79: 134–143. 16413248 10.1016/j.clpt.2005.10.005

[bib20] Hoey SE, Williams RJ, Perkinton MS. Synaptic NMDA receptor activation stimulates alpha-secretase amyloid precursor protein processing and inhibits amyloid-beta production. J Neurosci 2009; 29: 4442–4460. 19357271 10.1523/JNEUROSCI.6017-08.2009 PMC6665739

[bib21] Xia P, Chen HS, Zhang D, Lipton S A. Memantine preferentially blocks extrasynaptic over synaptic NMDA receptor currents in hippocampal autapses. J Neurosci 2010; 30: 11246–11250. 20720132 10.1523/JNEUROSCI.2488-10.2010 PMC2932667

[bib22] Bordji K, Becerril-Ortega J, Buisson A. Synapses, NMDA receptor activity and neuronal A*β* production in Alzheimer's disease. Rev Neurosci 2011; 22: 285–294. 21568789 10.1515/RNS.2011.029

[bib23] Knobloch M, Konietzko U, Krebs DC, Nitsch RM. Intracellular Abeta and cognitive deficits precede beta-amyloid deposition in transgenic arcAbeta mice. Neurobiol Aging 2007; 28: 1297–1307. 16876915 10.1016/j.neurobiolaging.2006.06.019

[bib24] Zempel H, Thies E, Mandelkow E, Mandelkow EM. Abeta oligomers cause localized Ca(2+) elevation, missorting of endogenous Tau into dendrites, Tau phosphorylation, and destruction of microtubules and spines. J Neurosci 2010; 30: 11938–11950. 20826658 10.1523/JNEUROSCI.2357-10.2010 PMC6633549

[bib25] De Felice FG, Wu D, Lambert MP, Fernandez SJ, Velasco PT, Lacor PN et al. Alzheimer's disease-type neuronal tau hyperphosphorylation induced by Abeta oligomers. Neurobiol Aging 2008; 29: 1334–1347. 17403556 10.1016/j.neurobiolaging.2007.02.029 PMC3142933

[bib26] Tanabe M, Gähwiler BH, Gerber U. Effects of transient oxygen-glucose deprivation on G-proteins and G-protein-coupled receptors in rat CA3 pyramidal cells *in vitro*. Europ J Neurosci 1998; 10: 2037–2045. 10.1046/j.1460-9568.1998.00215.x 9753091

[bib27] Tamburri A, Dudilot A, Licea S, Bourgeois C, Boehm J. NMDA-receptor activation but not ion flux is required for amyloid-beta induced synaptic depression. Plos One 2013; 8: e65350. 23750255 10.1371/journal.pone.0065350 PMC3672194

[bib28] Lynch G, Larson J, Kelso S, Barrionuevo G, Schottler F. Intracellular injections of EGTA block induction of hippocampal long-term potentiation. Nature 1983; 305: 719–721. 6415483 10.1038/305719a0

[bib29] Jensen L E, Bultynck G, Luyten T, Amijee H, Bootman MD, Roderick HL et al. Alzheimer's disease-associated peptide A*β*42 mobilizes ER Ca(2+) via InsP3R-dependent and -independent mechanisms. Front Mol Neurosci 2013; 6: 36. 24204331 10.3389/fnmol.2013.00036 PMC3817845

[bib30] Zhu Y, Pak D, Qin Y, McCormack SG, Kim MJ, Baumgart JP et al. Rap2-JNK removes synaptic AMPA receptors during depotentiation. Neuron 2005; 46: 905–916. 15953419 10.1016/j.neuron.2005.04.037

[bib31] Collingridge GL, Peineau S, Howland JG, Wang YT. Long-term depression in the CNS. Nat Rev Neurosci 2010; 11: 459–473. 20559335 10.1038/nrn2867

[bib32] Huang CC, You JL, Wu MY, Hsu KS. Rap1-induced p38 mitogen-activated protein kinase activation facilitates AMPA receptor trafficking via the GDI.Rab5 complex. Potential role in (S)-3,5-dihydroxyphenylglycene-induced long term depression. J Biol Chem 2004; 279: 12286–12292. 14709549 10.1074/jbc.M312868200

[bib33] Yang L, Mao L, Tang Q, Samdani S, Liu Z, Wang JQ et al. A novel Ca2+-independent signaling pathway to extracellular signal-regulated protein kinase by coactivation of NMDA receptors and metabotropic glutamate receptor 5 in neurons. J Neurosci 2004; 24: 10846–10857. 15574735 10.1523/JNEUROSCI.2496-04.2004 PMC6730215

[bib34] Renner M, Lacor PN, Velasco PT, Xu J, Contractor A, Klein WL et al. Deleterious effects of amyloid beta oligomers acting as an extracellular scaffold for mGluR5. Neuron 2010; 66: 739–754. 20547131 10.1016/j.neuron.2010.04.029 PMC3111138

[bib35] Hu N W, Nicoll AJ, Zhang D, Mably AJ, O'Malley T, Purro SA et al. mGlu5 receptors and cellular prion protein mediate amyloid-*β*-facilitated synaptic long-term depression *in vivo*. Nat Commun 2014; 5: 3374. 24594908 10.1038/ncomms4374 PMC4354159

[bib36] Harada J, Sugimoto M. An inhibitor of p38 and JNK MAP kinases prevents activation of caspase and apoptosis of cultured cerebellar granule neurons. Japn J Pharmacol 1999; 79: 369–378. 10.1254/jjp.79.369 10230866

[bib37] McLaughlin B, Pal S, Tran MP, Parsons AA, Barone FC, Erhardt JA et al. p38 activation is required upstream of potassium current enhancement and caspase cleavage in thiol oxidant-induced neuronal apoptosis. J Neurosci 2001; 21: 3303–3311. 11331359 10.1523/JNEUROSCI.21-10-03303.2001 PMC3746747

[bib38] Lipton SA. Paradigm shift in neuroprotection by NMDA receptor blockade: memantine and beyond. Nat Rev Drug Discov 2006; 5: 160–170. 16424917 10.1038/nrd1958

[bib39] Figueiredo CP, Clarke JR, Ledo JH, Ribeiro FC, Costa CV, Melo HM et al. Memantine rescues transient cognitive impairment caused by high-molecular-weight a*β* oligomers but not the persistent impairment induced by low-molecular-weight oligomers. J Neurosci 2013; 33: 9626–9634. 23739959 10.1523/JNEUROSCI.0482-13.2013 PMC6619709

[bib40] Scheff SW, Price D A, Schmitt FA, DeKosky ST, Mufson EJ. Synaptic alterations in CA1 in mild Alzheimer disease and mild cognitive impairment. Neurology 2007; 68: 1507–1508. 10.1212/01.wnl.0000260698.46517.8f 17470753

[bib41] Schneider LS, Dagerman KS, Higgins JP, McShane R. Lack of evidence for the efficacy of memantine in mild Alzheimer disease. Arch Neurol 2011; 68: 991–998. 21482915 10.1001/archneurol.2011.69

[bib42] Stoppini L, Buchs PA, Muller D. A simple method for organotypic cultures of nervous tissue. J Neurosci Methods 1991; 37: 173–182. 1715499 10.1016/0165-0270(91)90128-m

[bib43] Klein WL. Abeta toxicity in Alzheimer's disease: globular oligomers (ADDLs) as new vaccine and drug targets. Neurochem Int 2002; 41: 345–352. 12176077 10.1016/s0197-0186(02)00050-5

